# Homocysteine levels in patients with coronary slow flow phenomenon: A meta-analysis

**DOI:** 10.1371/journal.pone.0288036

**Published:** 2023-07-07

**Authors:** Hong Yu, Bei-Bei Wang, Meng Zhao, Feng Feng, Hua-Dong Li

**Affiliations:** 1 Department of Otorhinolaryngology, Union Hospital, Tongji Medical College, Huazhong University of Science and Technology, Wuhan, 430022, China; 2 School of Life Sciences, Westlake University, Hangzhou, 310024, China; 3 Department of Cardiology, The First People’s Hospital of Jinzhong, Jinzhong, 030699, China; 4 Institute of Biology, Westlake Institute for Advanced Study, Hangzhou, 310024, China; 5 Institute of Physical Education, Inner Mongolia Normal University, Hohhot, 010000, China; 6 Department of Cardiovascular Surgery, Union Hospital, Tongji Medical College, Huazhong University of Science and Technology, Wuhan,430022, China; The University of Mississippi Medical Center, UNITED STATES

## Abstract

**Background:**

With the development of coronary angiography, more and more attention has been paid to coronary slow flow phenomenon (CSFP). Recent studies have found that the correlation between homocysteine (Hcy) levels and CSFP was contradictory, so we conducted this meta-analysis to investigate the correlation.

**Methods:**

By March 2022, studies that meet the research requirements were identified by searching multiple databases including Embase, Web of Science, and PubMed. We included studies evaluating the correlation between Hcy levels and CSFP. Random or fixed effect meta-analyses were performed according to heterogeneity among included studies. A leave-out method and subgroup analyses were conducted to determine the source of heterogeneity.

**Results:**

Thirteen studies involving 625 CSFP and 550 subjects were included. After pooling data from each study, Hcy levels were higher in the CSFP groups (standard mean difference [SMD], 1.45; 95% CI, 0.94 to 1.96, *P* < .00001) than in the control group. In the meta-analysis, there was significant heterogeneity (*I*^2^ = 93%), which was further explored through leave-out method and and subgroup analyses. Specifically, pooling data from studies with a mean thrombolysis in myocardial infarction (TIMI) frame count ≥ 46 (SMD, 1.31; 95% CI, 1.00 to 1.63, *P* < .00001) resulted in no heterogeneity (0%), indicating that the TIMI frame count ≥ 46 was the source of heterogeneity.

**Conclusions:**

Our study found that elevated Hcy levels are strongly associated with CSFP. More importantly, the association was stronger in CSFP patients with mean TIMI frame count ≥ 46.

## Introduction

In 1972, Tambe et al. [1] first described coronary slow flow phenomenon (CSFP), an important angiographic feature characterized by delayed progression of contrast media injected through the coronary tree, which is seen in coronary syndromes. This phenomenon usually causes recurrent chest pain associated with myocardial ischemia or arrhythmia. It also leads to the adverse prognosis of myocardial infarction, ventricular remodeling, and increases the risk of major adverse cardiac events, including ischemic cardiomyopathy, congestive heart failure, and sudden cardiac death [2]. With the extensive development of coronary angiography, more such patients are found. The incidence of CSFP after coronary angiography in patients with clinically suspected coronary heart disease was 5.5–34.0% [3].

Although CSFP has been discovered for nearly 40 years, the pathophysiological mechanism of this phenomenon remains unclear. It is currently believed that its pathological mechanism may be related to inflammatory reaction, reperfusion, microcirculation disorder, atherosclerosis, and endothelial dysfunction. Homocysteine (Hcy), a sulfhydryl-containing amino acid, can cause endothelial dysfunction and generate free radicals, which induce oxidative stress [4]. Several studies [5,6] have shown that high Hcy levels are closely associated with endothelial dysfunction and oxidative stress, which increase cardiovascular risk regardless of classical risk factors. Some studies have found elevated plasma Hcy levels in asymptomatic and symptomatic adults with normal coronary arteries but coronary flow [7–9]. However, Mukhopadhyay et al [10] found there was no difference in Hcy levels between the CSFP group and the normal group (*P* > .05). Although extensive research has been conducted on this topic, no meta-analysis has been conducted to pool current data. To clarify the available epidemiological evidence, we systematically reviewed published literature and conducted this meta-analysis for the association of Hcy levels with CSFP.

## Materials and methods

### Search strategy

This meta-analysis was based on the Preferred Reporting Items for Systematic Reviews and Meta-Analysis Guidelines [11]. Two authors (HY and HDL) searched Embase, Web of Science, and PubMed databases for relevant studies published before March 2022. A literature search was conducted using the keywords "coronary slow flow", "coronary slow flow phenomenon", "CSFP" or "CSF" in combination with "homocysteine", "hyperhomocysteinemia", "homocysteine acid" or "Hcy". In addition, references to relevant studies were found for additional publications. Searches are limited to literature published in English. When the full article was not available, HY attempted to contact the corresponding author for more information. If there is any disagreement during the retrieval process, it can be resolved through consultation with the third author (FF).

### Inclusion criteria

Studies that met the following inclusion criteria included (1) comparative studies in humans; (2) reported plasma or serum Hcy levels in CSFP and controls; and (3) mean values with 95% confidence intervals (CI) were either directly extracted or indirectly calculated. (4) CSFP was quantified using thrombolysis in myocardial infarction (TIMI) frame count method [12,13].

### Exclusion criteria

Studies that met the following criteria were excluded (1) studies without CSFP research; (2) studies with duplicate data; (3) studies without available data; (4) studies on Hcy levels severely affected by pathological factors.

### Data extraction

Two authors (HY and HDL) independently extracted data from each included study, and a third author (FF) discussed and resolved differences in eligibility during the extraction process. The first author, year of publication, country, CSFP, and control group participants, matching factors, mean age, proportion of women, Body Mass Index (BMI), mean TIMI frame count, and TIMI frame count of right coronary artery (RCA), left circumflex artery(LCX), and left anterior descending artery(LAD) were extracted. The primary outcome of the study explored the association between Hcy levels and CSFP.

### Assessment of methodology quality

The modified Newcastle-Ottawa Scale (NOS) was used to assess the quality of included studies [14]. Studies with ≥6 points were considered to be of high quality.

### Statistical analysis

Standard mean difference (SMD) and corresponding 95% CI of Hcy levels for each study were pooled. The *I*^2^ test was used to assess statistical heterogeneity among included studies. When *I*^2^ ≥ 50% and *P* < 0.1, a random-effect method was performed to pool results together. Otherwise, a fix-effect method was performed. Subgroup analyses were performed on the basis of age, women, BMI, matching factors, and mean TIMI frame count and TIMI frame count of RCA, LCX, and LAD. This meta-analysis was conducted using the Review Manager software (RevMan 5.4). There is statistical significance when the *p-value* is less than 0.05.

The robustness of the results is evaluated by sensitivity analysis. Two different methods were used for this meta-analysis. First, meta-analysis was carried out by deleting each study one by one, i.e. a leave-one-out method. Second, subgroup analyses were conducted to explore potential heterogeneity. To assess potential publication bias, the Egger test was performed.

## Results

### Literature search

**Fig 1:**
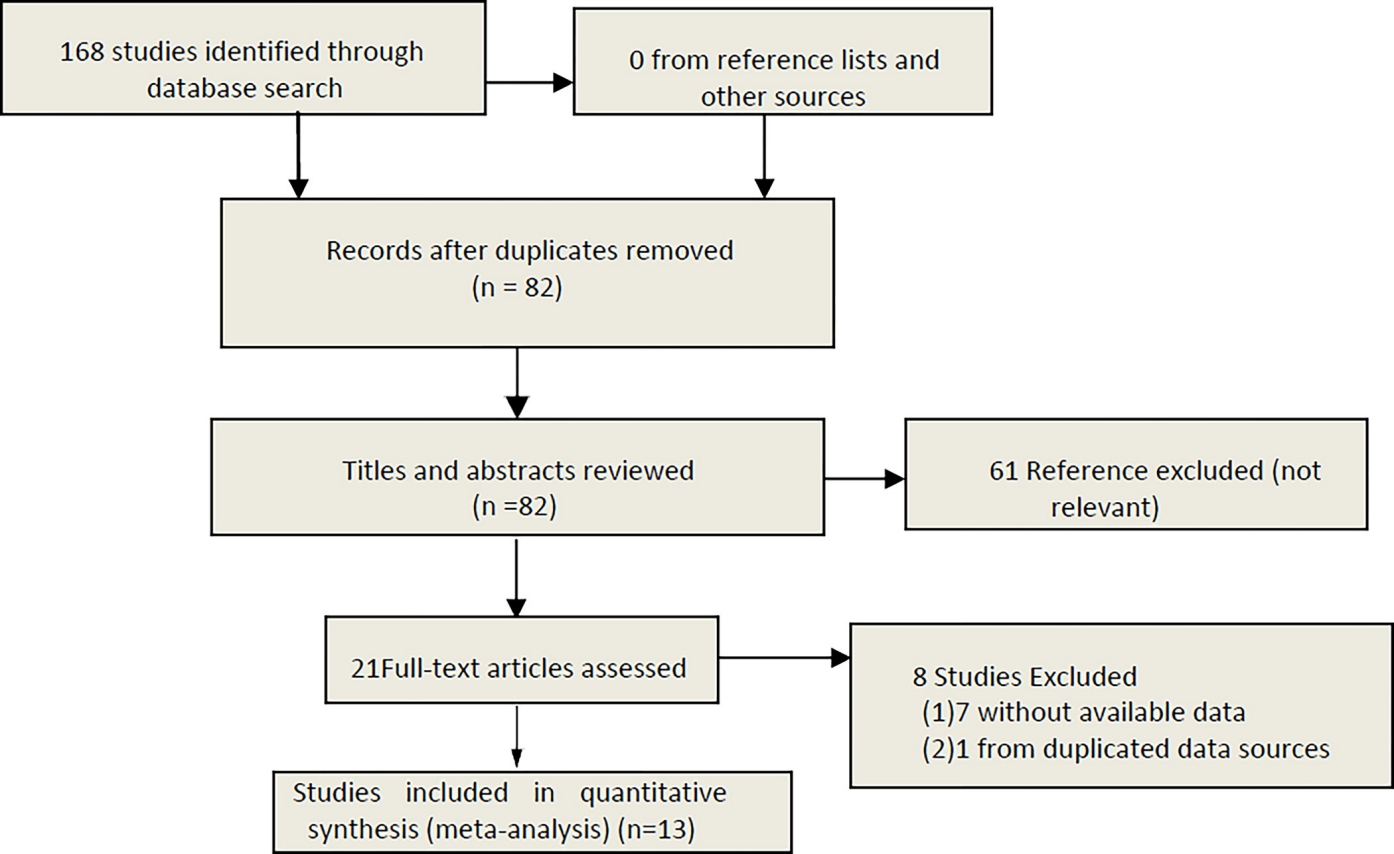
Study screening flow chart.

Of 168 studies (53 from Embase, 68 from Web of Science, and 47 from PubMed) identified from multiple databases, 86 duplicates were excluded. Sixty-one studies were excluded after reviewing the titles and abstracts of the remaining 82 studies. Twenty-one full-text studies were then evaluated. A total of 8 studies (7 without available data and 1 from duplicated data sources) were excluded. In addition, there were no identified studies from the reference list and other sources. Ultimately, a total of 13 studies [4,10,15–25] were included in our meta-analysis. The PRSIMA flow chart is shown in Fig 1.

### Characteristics of the included studies

In this meta-analysis, there were 13 studies involving 625 CSFP and 550 subjects. Hcy levels were measured using unknown in 2 studies, serum in 2 studies, and plasma in the remaining studies. All case-control studies were published in English from 2005 to 2022. Of the 13 studies, 8 were conducted in Turkey, 2 in China,1 in Australia, 1 in India, and 1 in Korea. Age, gender, BMI, blood pressure, and dyslipidemia were matched in most studies. Several comparable factors were also matched in several studies. The characteristics of the included studies are shown in Tables 1 and 2.

**Table 1:** Description of included studies.

Study	Year	Country	Study design	Sample	CSFP	Controls	Matched variable*	Study quality
**Tanriverdi [4]**	2007	Turkey	CC	Plasma	44 subjects with angiographically proven normal coronary arteries and CSFP in all three coronary vessels	44 subjects who had the same age and cardiovascular risk profile with angiographically proven normal coronary arteries without associated CSFP	1,2,3,4,5,6,7,8	6
**Mukhopadhyay [10]**	2017	India	CC	NA	40 consecutive patients with CSFP	40 controls with normal coronary flow	1,2,3,4,5,6,7	6
**Barutcu [15]**	2005	Turkey	CC	Plasma	39 patients with angiographically diagnosed CSFP but otherwise normal epicardial coronary arteries	30 subjects with angiographically normal coronary arteries	1,2,3,4	7
**Demirci [16]**	2019	Turkey	CC	Plasma	23 angiographically identified patients with normal coronary arteries and CSFP	25 angiographically normal coronary flow patients with a similar risk profile and demographic characteristics	1,2,3,4,5,6,7,8	6
**Evrengul [17]**	2006	Turkey	CC	Plasma	44 subjects with angiographically proven normal coronary arteries and CSFP in all three coronary vessels	44 subjects, matched for age and risk profile, with angiographically proven normal coronary arteries but without associated CSFP	1,2,3,4,5,6,7,8	6
**Evrengul [18]**	2007	Turkey	CC	Plasma	43 subjects with angiographically proven normal coronary arteries and CSFP in all three coronary vessels	43 subjects in the same age range and cardiovascular risk profile with angiographically proven normal coronary arteries and coronary flow	1,2,3,4,5,6,7,8	8
**Kopetz [19]**	2012	Australia	CC	Plasma	40 established CSFP	30 age-matched healthy controls	1,2,3,4,5,7	5
**Erbay [20]**	2005	Turkey	CC	Plasma	53 patients with angiographically proven normal coronary arteries and CSFP	50 subjects with angiographically proven normal coronary arteries without associated CSFP	1,2,3,4,5,6,7	7
**Tang [21]**	2014	China	CC	Serum	50 CSFP patients	25 controls	1,2,4,5,6,7	5
**Yang [22]**	2022	China	CC	Serum	73 patients with CSFP	73 controls with normal coronary flow	1,2,3,4,5,6,7	7
**Yoon [23]**	2012	Korea	CC	NA	41 patients with slow flow	41 patients without slow flow	1,2,3,4,5,6,7,8	8
**Yucel [24]**	2012	Turkey	CC	Plasma	50 patients with CSFP	An age- and gender-matched control group was composed of 30 patients with normal coronary arteries and normal coronary flow on coronary angiography	1,2,3,4,5,6,7,8	6
**Yurtdas [25]**	2013	Turkey	CC	Plasma	CSFP was determined in 41 of the 217 patients	41 subjects matched for demographic characteristics	1,2,3,4,5,6,7,8	7

CSFP: Coronary slow flow phenomenon; CNF: Normal coronary flow; NA: Not available; CC: Case-control

^†^ Study quality is evaluated by Newcastle-Ottowa Scale (1–9 stars).

*The matching factors:1. Age,2. Gender,3. Body mass index, 4. Blood pressure,5. Dyslipidemia,6. Diabetes mellitus, 7. Smoking,8. Family history of coronary heart disease.

**Table 2:** Participant characteristics.

Study	Mean (SD) age, y CSF CNF		Women, sex, n (%) CSF CNF		Mean (SD) BMI,Kg/m^2^ CSF CNF	
**Tanriverdi [4]**	55.5(10.4)	53.9(11)	18(41)	22(50)	NA	NA
**Mukhopadhyay [10]**	50.43(10.18)	51.38(7.19)	13(7.5)	15(12.5)	27.27(2.82)	24.12(2.35)
**Barutcu [15]**	47(8)	46(8)	13(33.3)	10(33.3)	26(3)	25(3)
**Demirci [16]**	50.5(11.1)	53.7(10.0)	10(43.5)	16(64)	28.6(5.07)	29.1(2.93)
**Evrengul [17]**	55.5(10.4)	53.9(11)	18(40.9)	22(50)	NA	NA
**Evrengul [18]**	56.3(10.6)	53.1(10.6)	17(39.5)	20(46.5)	29.5(3.7)	28.8(3.4)
**Kopetz [19]**	55(9.6)	56(7.4)	10(44)	15(38)	30(1.0)	27(0.9)
**Erbay [20]**	48(9)	50(8)	22(44)	21(40)	NA	NA
**Tang [21]**	61.74(9.4)	64.48(8.24)	20(40)	18(72)	NA	NA
**Yang [22]**	59.30(12.50)	60.50(10.20)	32(44)	30(41)	25.22(3.09)	24.63(2.33)
**Yoon [23]**	56.3(10)	56.1(8)	22(50)	21(51.3)	25.21(3.5)	25.06(3.1)
**Yucel [24]**	53.0(9.1)	51.1(8.1)	23(46)	15(50)	NA	NA
**Yurtdas [25]**	49(8)	50(9)	13(32)	18(44)	27.4(5.4)	26.1(4.7)

**Study**	**LAD** **CSFP CNF**		**LCX** **CSFP CNF**		**RCA** **CSFP CNF**		**Mean TIMI frame count** **CSFP CNF**	
**Tanriverdi [4]**	61.9(7.2)	29.1(5.5)	37(7.1)	20.1(3.3)	38.7(6.6)	19.6(3.9)	45.9(12)	23.3(3.7)
**Mukhopadhyay [10]**	38.47(9.4)	17.69(2.65)	39.4(11.2)	17.72(2.0)	35.6(6.8)	18.2(2.3)	NA	NA
**Barutcu [15]**	61.2(7.4)	34.2(2.3)	39.4(4.3)	23.4(3.5)	38.2(4.8)	24.3(4.5)	NA	NA
**Demirci [16]**	46.9(3.0)	20.1(1.9)	38.8(4.9)	18.4(1.2)	38.8(4.8)	19.2(1.4)	NA	NA
**Evrengul [17]**	61.9(7.2)	29.1(5.5)	37(7.1)	20.1(3.3)	38.7(6.6)	19.6(3.9)	45.9()12	23.2(3.7)
**Evrengu [18]**	62.8(11.2)	28.9(5.6)	36.3(10.5)	21.2(3.2)	39.8(6.6)	19.8(3.8)	46.3(8.7)	24.3(2.9)
**Erbay [20]**	51(12)	27(6)	46(8)	26(7)	47(11)	27(8)	48(9)	27(6)
**Tang [21]**	60.8(17.5)	35.4(6.3)	46.3(11.6)	24.1(2.5)	37.2(11)	20.4(4)	39.8(9.6)	21.8(2.7)
**Yucel [24]**	32.2(8.7)	15,7(1.8)	27.0(9)	18.5(2.9)	20.7(4.6)	16.5(2.6)	26.8(5.5)	16.9(2.0)
**Yurtdas [25]**	50(12)	27(4)	38(13)	24(3)	37(12)	22(3)	42(7)	24(2)

CSFP: Coronary slow flow phenomenon; CNF: Normal coronary flow; SD: Standard Deviation; NA: Not available; BMI: Body mass index. LAD: Left anterior descending artery; LCX: Left circumflex artery; RCA: Right coronary artery; TIMI: Thrombolysis in myocardial infarction.

### Quality assessment

Since all included studies were case-control studies, the methodological quality of each included study was assessed through NOS. Of the 13 studies, two scored less than 6 points. Five points in two studies and the eight points in two studies were the lowest and highest scores, respectively.

### Main results

**Fig 2:**
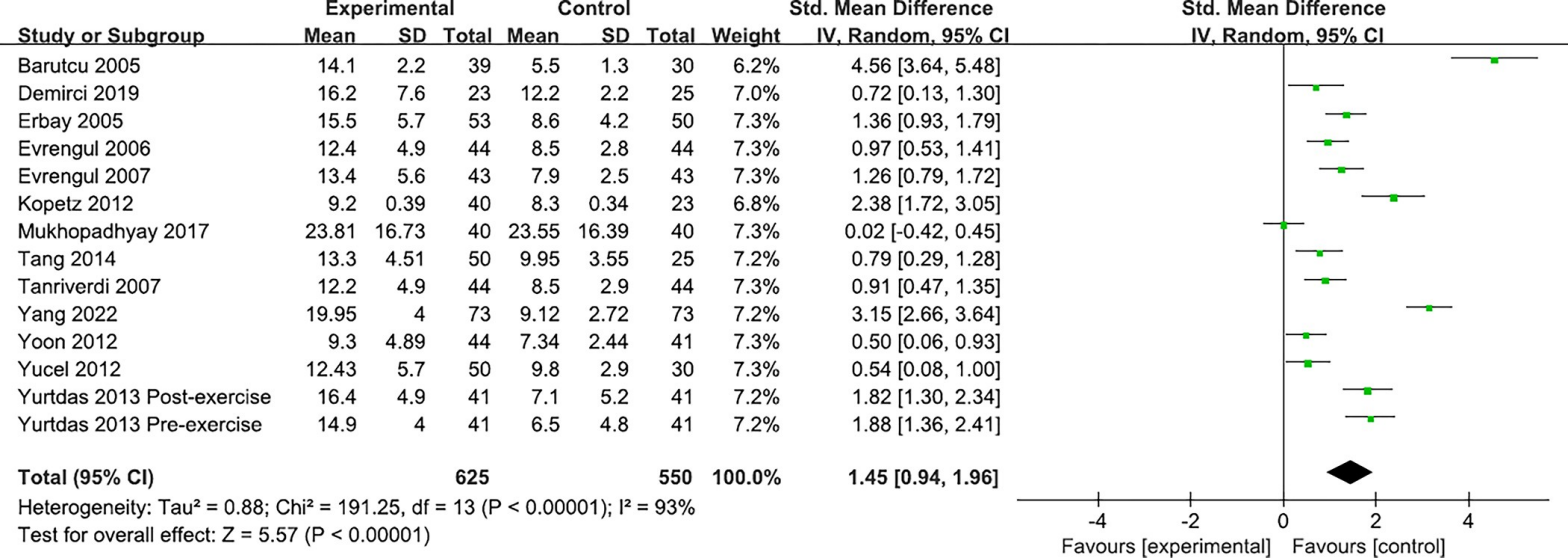
Forest plot of the impact of the Hcy levels on CSFP. SMD, standard mean difference; CSFP, coronary slow flow phenomenon; Hcy, homocysteine.

The main results of this meta-analysis are shown in **Fig 2**. Compared to the control group, Hcy levels were higher in the CSFP groups(SMD, 1.45; 95% CI, 0.94 to1.96, *P* < .00001). Due to the significant heterogeneity among the studies (*I*^2^ = 93%), the random model was adopted. Subgroup analyses were performed based on age, women, BMI, matching factors, TIMI frame count of LAD, LCX, RCA and mean TIMI frame count (Table 3).

**Table 3:** Results of subgroup analysis among CSF vs CHF.

Subgroup	Studies included(N)	Sample Size(CSX/CG)	Chi Square(*df*)	*P* value	Pooledoverall SMD (95%)	Heterogeneity(*I*^*2*^)
**Age<55(years)**	6	287/257	102.7(6)	0.0003	1.50 [0.69, 2.32]	94
**Age≥55(years)**	7	338/293	87.54(6)	**<**.0001	1.41 [0.72, 2.10]	93
**Women<50%**	12	581/509	178.96(12)	**<**.00001	1.53 [0.99, 2.07]	93
**Women≥50%**	1	44/41	NA	0.02	0.50 [0.06, 0.93]	NA
**BMI<26Kg/m** ^**2**^	2	177/114	63.41(1)	0.17	1.82 [-0.78, 4.42]	98
**BMI≥26Kg/m** ^**2**^	6	267/243	103.67(6)	**<**.0001	1.76 [0.89, 2.63]	94
**matched variables<8**	6	295/245	145.36(5)	**<**.00001	2.01 [0.84, 3.18]	97
**matched variables = 8**	7	330/309	31.78(7)	0.002	1.07 [0.71,1.43]	78
**TIMI frame count for LAD<60**	4	198/197	42.63(4)	0.0003	1.16 [0.44, 1.88]	91
**TIMI frame count for LAD≥60**	5	220/186	57.04(5)	**<**.00001	1.61 [0.75, 2.47]	93
**TIMI frame count for LCX<38**	4	181/161	4.73(3)	0.0002	0.92 [0.69, 1.14]	37
**TIMI frame count for LCX≥38**	6	287/252	97.55(6)	0.006	1.54 [0.74, 2.34]	94
**TIMI frame count for RCA<38**	4	222/177	43.92(4)	**<**.0001	1.00 [0.28, 1.72]	91
**TIMI frame count for RCA≥38**	5	246/236	57.36(5)	0.0004	1.54 [0.84, 2.25]	91
**Mean TIMI frame count<46**	5	182/137	24.37(5)	**<**.00001	1.14[0.71, 1.57]	79
**Mean TIMI frame count≥46**	2	96/93	0.11(1)	**<**.00001	1.31 [1.00, 1.63]	0

CSFP: Coronary slow flow phenomenon; CNF: Normal coronary flow; SD: Standard Deviation; NA: Not available; BMI: Body mass index. LAD: Left anterior descending artery; LCX: Left circumflex artery; RCA: Right coronary artery; TIMI: Thrombolysis in myocardial infarction.

### Heterogeneity, sensitivity analysis, and publication bias

There was significant heterogeneity in the meta-analysis, and subgroup analysis found that mean TIMI frame count ≥ 46 was the source of heterogeneity (Table 3). When each study was sequentially removed from the included studies and meta-analysis was performed again, the overall results remained the same. After excluding two low-quality studies, Hcy levels were found to be higher in the CSFP group (SMD: 1.43.95% CI: 0.87 to2.00, *P***<**.00001) than in the control group. Moreover, after excluding studies with matching factors≥8, a significant association was still observed between the two groups (SMD:2.01,95%CI:0.84 to3.18, *P* = .0007). The Egger test (*P*>.05) showed that there was no potential publication bias in Hcy levels between CSFP and control groups.

## Discussion

Numerous studies have focused on the relationship between Hcy levels and CSFP, but the results have been inconsistent. Our first meta-analysis found a significant relationship between elevated Hcy levels and CSFP. However, a value of *I*^2^ = 93% (*I*^2^>50%, *P* < .01) indicated a high degree of heterogeneity among studies. Therefore, we conducted a subgroup analysis and found that the mean TIMI frame count≥46 was the source of heterogeneity. In addition, sensitivity analyses were performed to confirm robust results. Egger’s test (*P*>.05) found no potential publication bias. Therefore, we are confident in the data obtained in our meta-analysis, which showed that there is a close correlation between high Hcy levels and CSFP.

Some potential confounding factors, such as age, sex, and BMI cannot be ignored as they are strongly associated with both elevated Hcy levels and CSFP. Age, gender, and BMI are matched in most studies. Weather data from studies with 8 matched variables or < 8 matched variables were pooled analysis, and the pooled result was consistent. Therefore, we believe that these confounding factors are evenly distributed in the case group and control group. Potential confounding factors did not affect the reliability of the results.

Current studies suggest that Hcy-induced endothelial cell damage and microcirculation dysfunction play an important role in CSFP. Elevated Hcy levels may induce high oxidative stress, which increases the production of oxygen radicals [26]. Oxygen free radicals can reduce the nitric oxide (NO) production by inhibiting nitric oxide synthase, resulting in serious damage to vascular endothelial cells [27]. Elevated Hcy levels may also affect NO synthesis and thrombin regulatory protein activity by enhancing the automatic oxidation of low density lipoprotein, resulting in further damage to endothelial cell damage [28]. High homocysteine levels may induce the expression of cyclins D and A, which can stimulate vascular smooth muscle proliferation and increase vascular resistance [29]. High homocysteine levels may accelerate the expression of thrombomodulin, von Willebrand factor, and cell adhesion molecules. They can cause damage to vascular endothelial cells, promote smooth muscle cell proliferation, and ultimately increase vascular resistance [25,27,30,31].

Our study found a close relationship between elevated Hcy levels and CSFP, which was consistent with Li et al. [32] Since previous studies found that age, gender, BMI, and TIMI frame count were closely related to Hcy levels and CSFP, we performed a subgroup analysis on them. Hcy levels increase with age, and the decline of liver and kidney function in the elderly leads to the reduction of Hcy metabolism, which increases the serum Hcy concentration [33,34]. The digestive and absorption dysfunction of the elderly leads to the deficiency of vitamin B12 and folic acid, which affects the metabolism of Hcy levels [34]. Our result showed elevated Hcy levels were significantly associated with CSFP in people aged < 55 years and ≥ 55 years. The main reason is that the number of included studies is small and the age of the included population is mainly between 50 and 60 years. Elevated Hcy levels were significantly associated with CSFP in women <50% and Women ≥50%. Because only one of the included studies accounted for 50% of women, while the proportion of women in the other studies was less than 50%, women played a small role in elevated Hcy levels and CSFP. Tang [22] et al. found that CSFP was associated with high plasma Hcy levels, and men were more vulnerable to CSFP, consistent with our findings. Men need to produce creatine more frequently than women because they have more muscle mass. Part of the methyl donor required for creatine synthesis comes from the conversion of S-adenosine methionine to S-adenosine homocysteine. S-adenosine homocysteine is the precursor of Hcy. Instead, studies have shown that women have a greater Hcy flux through the sulfur-conversion pathway, which reduces Hcy concentrations [35,36]. Elevated Hcy levels were significantly associated with CSFP in BMI ≥ 26kg/m2, but this association was not significant in BMI<26kg/ m^2^, which was similar to the results of Mukhopadhyay et al [10]. A meta-analysis, conducted in 2020, showed that obese patients tended to have higher Hcy levels [37], resulting in CSFP. In additional, Obesity has been shown to be independently associated with coronary systemic endothelial dysfunction [38]. Three coronary arteries were associated with high Hcy levels both lower TIMI frame counts(LAD**<**60, LCX**<**38 and RCA**<**38) and higher TIMI frame counts(LAD≥60, LCX≥38 and ≥38), but the association was stronger at higher TIMI frame counts. We also found that mean TIMI frame counts ≥ 46 were more closely related to elevated Hcy levels than mean TIMI frame counts < 46. When we pooled data from studies with mean TIMI frame count ≥ 46, the heterogeneity was found to be 0, so we considered mean TIMI frame count ≥ 46 as the source of heterogeneity. Therefore, we showed that there was a strong association between elevated Hcy levels and CSFP.

Although CSFP does not have obvious coronary stenosis, it can lead to myocardial insufficiency, myocardial ischemia, hypoxia, and even acute coronary syndrome, fatal arrhythmia, sudden cardiac death, and other malignant cardiovascular events, which place a burden on the patient’s family and society [39,40]. Therefore, early detection of CSFP risk factors has a positive effect on the prevention and treatment of CSFP. We used meta-analysis for the first time to demonstrate that elevated Hcy levels are strongly associated with CSFP. Therefore, we suggest that elevated Hcy levels should be considered as a novel risk factor for CSFP in future CSFP-related clinical guidelines. Large-scale, well-designed, multi-center clinical studies are needed to establish a reasonable cutoff of Hcy levels to prevent CSFP.

Several limitations of this meta-analysis are discussed here. First, although we explored sources of heterogeneity through sensitivity analyses, we were unable to detect heterogeneity elsewhere due to a lack of sufficient data. Second, the detection of serum or plasma Hcy levels by different instruments or methods may also lead to deviations in results. Third, there are not enough studies and samples, which may have an impact on the accuracy of the results. Therefore, larger studies should be conducted to confirm our study.

## Conclusions

Our study found that elevated Hcy levels are strongly associated with CSFP. More importantly, the association was stronger in CSFP patients with mean TIMI frame count ≥ 46.
